# Efficacy of exogenous pyruvate in Trembler^J^ mouse model of Charcot‐Marie‐Tooth neuropathy

**DOI:** 10.1002/brb3.1118

**Published:** 2018-09-21

**Authors:** Zarife Sahenk, Mehmet E. Yalvac, Jakkrit Amornvit, William David Arnold, Lei Chen, Kimberly M. Shontz, Sarah Lewis

**Affiliations:** ^1^ Center for Gene Therapy The Research Institute at Nationwide Children’s Hospital Columbus Ohio; ^2^ Department of Pediatrics and Neurology Nationwide Children’s Hospital and The Ohio State University Columbus Ohio; ^3^ Department of Pathology and Laboratory Medicine Nationwide Children’s Hospital Columbus Ohio; ^4^ Department of Neurology The Ohio State University Columbus Ohio; ^5^ King Chulalongkorn Memorial Hospital Chulalongkorn University Bangkok Thailand; ^6^ Department of Medicine, Faculty of Medicine Chulalongkorn University Bangkok Thailand; ^7^ Department of Physical Medicine and Rehabilitation The Ohio State University Columbus Ohio

**Keywords:** CMT neuropathies, exogenous pyruvate, improved nerve function, NT‐3

## Abstract

**Introduction:**

Classic Charcot‐Marie‐Tooth (CMT) neuropathies including those with Schwann cell genetic defects exhibit a length‐dependent process affecting the distal axon. Energy deprivation in the distal axon has been the proposed mechanism accounting for length‐dependent distal axonal degeneration. We hypothesized that pyruvate, an intermediate glycolytic product, could restore nerve function, supplying lost energy to the distal axon.

**Methods:**

To test this possibility, we supplied pyruvate to the drinking water of the Trembler‐J (Tr**^J^**) mouse and assessed efficacy based on histology, electrophysiology, and functional outcomes. Pyruvate outcomes were compared with untreated Tr**^J^** controls alone or adeno‐associated virus mediated NT‐3 gene therapy (AAV1.NT‐3)/pyruvate combinatorial approach.

**Results:**

Pyruvate supplementation resulted increased myelinated fiber (MF) densities and myelin thickness in sciatic nerves. Combining pyruvate with proven efficacy from AAV1.tMCK.NT‐3 gene therapy provided additional benefits showing improved compound muscle action potential amplitudes and nerve conduction velocities compared to pyruvate alone cohort. The end point motor performance of both the pyruvate and the combinatorial therapy cohorts was better than untreated Tr**^J^**controls. In a unilateral sciatic nerve crush paradigm, pyruvate supplementation improved myelin‐based outcomes in both regenerating and the contralateral uncrushed nerves.

**Conclusions:**

This proof of principle study demonstrates that exogenous pyruvate alone or as adjunct therapy in Tr**^J^** may have clinical implications and is a candidate therapy for CMT neuropathies without known treatment.

## 1 INTRODUCTION

Neuropathological studies of experimental toxic neuropathies from 1970s unveiled an important feature of axonal degeneration. The key observation was that during the evolution of “dying back” axonopathy, distal segments of long axons respond in a stereotypical manner under so many diverse and seemingly unrelated detrimental conditions. Disruption of energy‐dependent axonal transport system as the mechanism underlying this length‐dependent distal axonal degeneration, first proposed more than 30 years ago by Spencer, Sabri, Schaumburg, and Moore (1979) is now being revisited with renewed enthusiasm and a slightly different perspective, from an angle of glia‐axon interactions and the increasing awareness of the essential role of bioenergetics in axon maintenance (Beirowski et al., 2014; Brown, Evans, Black, & Ransom, 2012; Funfschilling et al., 2012; Lee et al., 2012; Nave, 2010; Viader et al., 2011).

Most CMT patients including those with primary Schwann cell (SC) genetic defects present with a clinical phenotype of length‐dependent axonal disease. Our previous studies and others have shown that axonal pathology in so called demyelinating Charcot‐Marie‐Tooth (CMT) neuropathies is an important feature that directly correlates with the clinical disability (Dyck, Lais, & Offord, 1974; Krajewski et al., 2000; Sahenk, 1999; Sahenk & Chen, 1998; Sahenk, Chen, & Mendell, 1999). Profound axonal cytoskeletal abnormalities leading to axonal degeneration and preferential distal axonal loss seen in Tr^J^ mice and in xenografts from patients with primary SC genetic defects were thought to result from impaired SC‐axon interactions (de Waegh & Brady, 1990, 1991 ; de Waegh, Lee, & Brady, 1992; Sahenk, 1999). Although the mechanism for such impairment is not yet fully understood, recent hypothesis suggests lack of trophic support from SCs for the mitochondrial energy metabolism in distal axons (Nave, 2010).

A series of seminal experiments in early 1970s had shown that fast axonal transport is closely dependent on oxidative phosphorylation (Ochs & Hollingsworth, 1971; Ochs & Ranish, 1970) and that the efficacy of axonal glycolysis is limited in a length‐dependent fashion (Sabri et al., 1989; Spencer et al., 1979). Inhibition of glyceraldehyde dehydrogenase (GADH) by iodoacetate was shown to perturb fast axonal transport as a function of declining levels of ATP and creatine phosphate. Moreover, pyruvate supplementation allowed the nerve to bypass the blockade in energy production pathways and restored axonal transport by providing alternative substrate of oxidative metabolism (Sabri & Ochs, 1971). Theoretically, if SC‐derived glycolysis products (pyruvate, lactate) provide trophic support for axonal mitochondria in maintaining long axons, exogenous pyruvate could be beneficial, particularly in dysmyelinating CMT neuropathies where axons are engulfed by the mutant, dysfunctional SCs. To test this possibility, we used Tr^J^ mouse model which carries a point mutation in peripheral myelin 22 (PMP22) gene and assessed the efficacy of exogenous pyruvate upon the histopathological features of intact and regenerating trembler sciatic nerves. We report here that exogenous sodium pyruvate added to drinking water in Tr^J^ mice increased myelinated fiber (MF) density and myelin thickness in the sciatic nerves, resulting improvements in functional and electrophysiological parameters. These observations have direct relevance to potential treatment value of pyruvate supplementation in peripheral neuropathies with diverse causes in considering the fact that clinically, sodium pyruvate has already been given to patients for a variety of conditions from Friedreich’s ataxia, mitochondrial disease to open heart surgery (Dijkstra et al., 1984; Fujii et al., 2014; Giannelli, McKenna, Bordiuk, Miller, & Jerome, 1976; Koga et al., 2012; Olivencia‐Yurvati, Blair, Baig, & Mallet, 2003; Tanaka et al., 2007) and acute heart failure (Hermann, 2001; Hermann et al., 1999). Moreover, we show in a CMT mouse model that combining pyruvate supplementation with the proven efficacy from adeno‐associated virus‐NT‐3 (AAV1.NT‐3) gene therapy enhanced peripheral nerve function and improved histology as proof of principal for a combinatorial treatment approach.

## 2 METHODS

### 2.1 Animals and treatment groups

Six‐ to eight–week‐old male Tr^J^ mice (*pmp22^Tr‐J^*) were used in this study. Tr^J^ mice carrying a spontaneous point mutation (Leu16Pro) in the myelin gene PMP22 (Suter et al., 1992) were obtained from the Jackson Laboratory (JAX Mice, Main. Stock No: 002504, http://scicrunch.org/resolver/SCR_002798). All treatment protocols and animal surgeries were conducted under the protocols approved by the Nationwide Children’s Hospital Animal Care and Use Committee. Animals were acclimatized by placing on 2.5% apple juice in drinking water (freshly prepared every other day from commercially available 100% apple juice) first, which was increased to 10% a week later. In pyruvate cohorts, sodium pyruvate (Sigma) was added to sweetened drinking water at 2.5% concentration and then increased to 4% final concentration in 7 days. Cohort 1 received pyruvate alone (*n* = 6); Cohort 2 (the combinatorial treatment group; *n* = 9), received, self‐complementary (sc) AAV1.tMCK.NT‐3 at 1 x 10^11^ vg, by intramuscular injection (30 µl) to the left gastrocnemius muscle, on the start day of pyruvate treatment. Vector was produced under the same conditions previously described, and aliquots were kept in −80°C until use (Sahenk et al., 2014). Cohort 3, the untreated trembler‐control group (*n* = 6) received 30 µl of intramuscular PBS injection and maintained on drinking water with 10% apple juice. End point NT‐3 serum levels were measured using ELISA as we previously shown (Sahenk et al., 2014).

### 2.2 Electrophysiology and functional studies

Sciatic motor nerve conduction studies were performed bilaterally in each cohort mice at baseline and at 16 weeks after the onset of treatments using an electrodiagnostic system (Synergy N2 electromyograph; Natus, Middletown, WI) as we and others previously reported (Yalvac et al., 2015, 2014). Briefly, the sciatic motor nerve conduction responses were recorded using two fine ring electrodes (Alpine Biomed, Skovlunde, Denmark) used as the active (E1) and reference (E2) electrodes. The active recording electrode was placed over the proximal portion of the gastrocnemius muscle and the reference electrode over the mid‐metatarsal region of the foot. Using an irrigating syringe, the ring electrodes were precisely coated with electrode gel (Spectra 360 by Parker laboratories, Fairfield, NJ) to reduced skin impedance. A pair of 28 gauge monopolar needle electromyography electrodes (Teca, Oxford Instruments Medical, New York, NY) was used to deliver supramaximal stimulus to the sciatic nerve at the distal thigh and sciatic notch. The parameters measured included compound muscle action potential (CMAP) amplitude, distal latency, and conduction velocity.

*Functional studies* included bilateral hind‐limb grip strength testing (Sahenk et al., 2014, 2010 ) and wire hanging test (four‐limb grip strength test) were performed at the end point on three consecutive days following previously described and established protocols in our Center (Yalvac et al., 2014).

### 2.3 Tissue allocation and histological analysis

All treatment groups were euthanized at 16 weeks of treatment period for histological and molecular studies. Both sciatic nerves were removed; one side was snap frozen for qPCR analysis. The other side in its in situ length was fixed in 3% glutaraldehyde in 0.1 M phosphate buffer and further processed for plastic embedding according to established methods in our laboratory. One‐micrometer thick cross sections from the plastic‐embedded mid‐sciatic nerves were used for qualitative and quantitative studies.

### 2.4 Surgical procedure for regeneration paradigm

Another set of experiments were used to test the efficacy of pyruvate supplementation on the trembler nerve regeneration. Total of eight Tr^J^ mice underwent sciatic nerve crush procedure 1 week after starting of 4% sodium pyruvate in apple juice (*n* = 5) or apple juice alone as control (*n* = 3). Under isoflurane anesthesia, left sciatic nerves were exposed and crushed with a fine forceps at a level 5 mm distal to the sciatic notch to generate a regeneration paradigm as we previously described (Sahenk et al., 2014, 2010 ). The crush site was marked by a 10‐0 nylon suture tie passed through the epineurium. Twenty weeks post‐crush, mice were killed quickly by an overdosage of xylazine/ketamine anesthesia, and the sciatic nerves from crushed and intact sites were removed. Approximately 2 mm in length tissue blocks immediately distal to the crush site and the subsequent three segments, all marked for proximo‐distal orientation as well as the mid‐sciatic segments from the contralateral uncrushed nerves were processed for plastic embedding.

### 2.5 Myelinated fiber density determinations

Quantitative analysis at the light microscopic level was performed on 1‐µm thick toluidine blue‐stained cross sections from mid‐sciatic nerve segments and the segments approximately 3 mm distal to the crush site in the regenerating sciatic nerves. Four randomly selected areas were photographed at 100× magnification, and axon diameter measurements were obtained from the computer screen image frames using BioQuant Life Sciences imaging software (BioQuant Image Analysis Corporation; Nashville, Tennessee). In Cohort 1 (the pyruvate only group; *n* = 6), a total of 3,415 and in Cohort 2 (the combinatorial treatment group; *n* = 6,), 3,595 measurements were made. In nerve crush experiments, a total of 1,106 of regenerating fibers and 1,271 of intact fibers from the contralateral sciatic nerve in the untreated control (*n* = 3) and 2,844 of regenerating fibers and 2,583 of intact and in the pyruvate group (*n* = 5) were measured. Composites of fiber size distribution histograms and mean MF densities (mean ±SEM, number/mm^2^) were generated.

### 2.6 G ratio of the myelinated fibers

The G ratio refers to the ratio of axonal diameter/fiber diameter, and lower g ratios represent axons with thicker myelin (Beuche & Friede, 1985; Friede & Beuche, 1985). For each animal (*n* = 3 in each group), measurements from all MFs in two randomly selected representative unit areas were included; a total of 569 measurements per untreated, 766 per pyruvate, and 905 per combinatorial treatment cohorts were obtained to generate the percent G ratio distribution histograms and scattergrams as previously described (Sahenk et al., 2014).

### 2.7 Quantitative real‐time PCR analysis

Total RNA was extracted from snap frozen sciatic nerve samples of treated and control Tr^J^ mice at 16‐week end point. RNA isolation from each sample was done by using mirVana RNA isolation kit (Life Technologies, #AM1560, TX), and subsequently, the cDNAs were synthesized by using Trascriptor First Strand cDNA synthesis kit (Roche, # 04379012001 Roche) following manufacturer’s instructions. qPCR experiments were performed by using iTaq™ universal SYBR^®^ Green supermix (Biorad, #1725122, Hercules, CA). Primer sequences for PGC1α (Cunningham et al., 2007) and GAPDH (Toscano et al., 2010; housekeeping gene) were found in the literature. Other primers sequences were obtained from Primer Bank (Wang et al., 2012)**.** All qPCR experiments were done by using ABI 7,500 real‐time PCR machine, and the results were analyzed using Data Assist Software (ABI).

### 2.8 Statistical analysis

GraphPad Prism software (La Jolla, CA; version number 7, http://scicrunch.org/resolver/SCR_002798) was used for all statistical analyses. Statistical difference was calculated by using Student’s *t* test or one‐way analysis of variance followed by Tukey's multiple comparison tests or regression analysis when applicable.

## 3 RESULTS

### 3.1 Electrophysiological and functional improvements with pyruvate treatment

**Figure 1:**
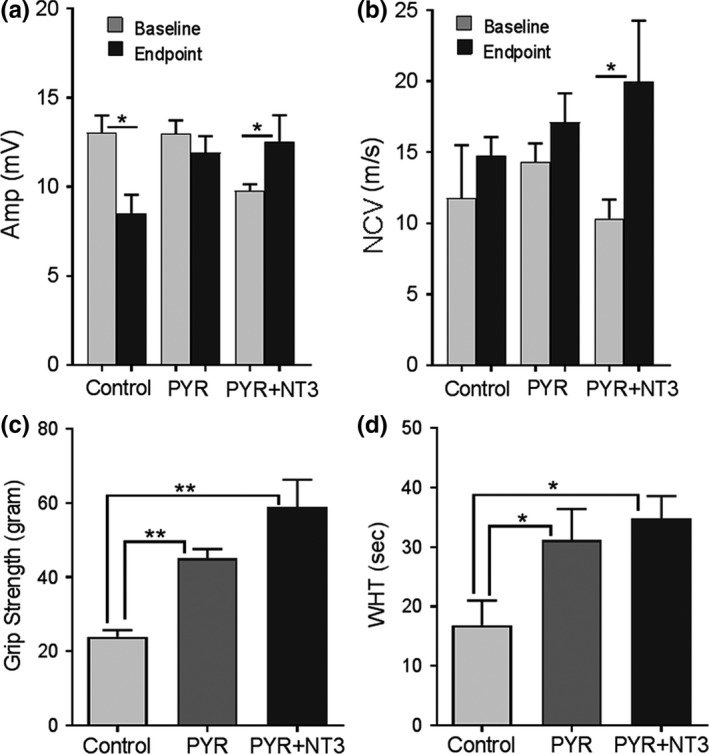
Sciatic nerve conduction studies in Tr**^J^** mice (a) performed at baseline and end point showed pyruvate (PYR) treatment preserved compound muscle action potential (CMAP) amplitudes during the 16 weeks of treatment period while a significant deterioration occurred in the untreated control Tr**^J^** group (*n* = 6 in each cohort). PYR and AAV1.NT‐3 combinatorial therapy (PYR + NT‐3; *n* = 9) significantly improved end point CMAPs and nerve conduction velocities (a and b) (Student’s *t‐* test; *p < 0.05). End point motor functions at 16 weeks were tested by performing hind‐limb grip strength (c) and four‐limb wire hanging test (d). Both PYR (*n* = 6) and PYR + NT‐3 group (*n* = 9) performed better than control group (*n* = 6) in both test (one‐way ANOVA for B and C; *p < 0.05, **p < 0.01). Error bars represent standard error of means

Sciatic nerve conduction studies performed at baseline and end point showed that the pyruvate‐treated Tr**^J^** mice (Cohort 1) preserved compound muscle action potentials (CMAPs) during the 16 weeks of treatment period while a significant deterioration occurred in the untreated control Tr**^J^** group (Cohort 3; *p* < 0.05; Figure 1a). The nerve conduction velocities and distal latency changes were not significantly different between these groups. On the other hand, we observed additive effects of pyruvate and AAV1.tMCK.NT‐3 combination therapy, which significantly improved CMAP amplitudes (9.12 ± 0.58 vs. 12.54 ± 1.47 mV, *n* = 9, *p* < 0.05) and the sciatic nerve conduction velocities (10.33 ± 1.34 vs. 19.98 ± 4.30; *p* < 0.05) at the end point compared to the baseline, even though this group at baseline started with lower parameters compared with others (Figure 1a,b). We confirmed the additive effects of the AAV1.NT‐3 gene therapy by illustrating the presence of significantly increased serum NT‐3 levels in the cohort receiving combinatorial therapy measured by ELISA at 16 weeks (Supporting Information Figure S1). Cohorts 1 and 3 did not show similar increases in serum NT‐3.

The end point motor performance (simultaneous bilateral hind‐limb grip strength and four‐limb wire hanging test) at 16 weeks revealed the efficacy of pyruvate supplementation in the Tr**^J^**mouse model (Figure 1c,d). Both the pyruvate and the pyruvate plus AAV1.tMCK.NT‐3 combinatorial therapy cohorts performed significantly better than the untreated control Tr^J^ group. Although the cohort receiving the combinatorial therapy performed slightly better than the pyruvate alone group (*p* = not significant).

### 3.2 Histopathological improvements in trembler nerve

**Figure 2:**
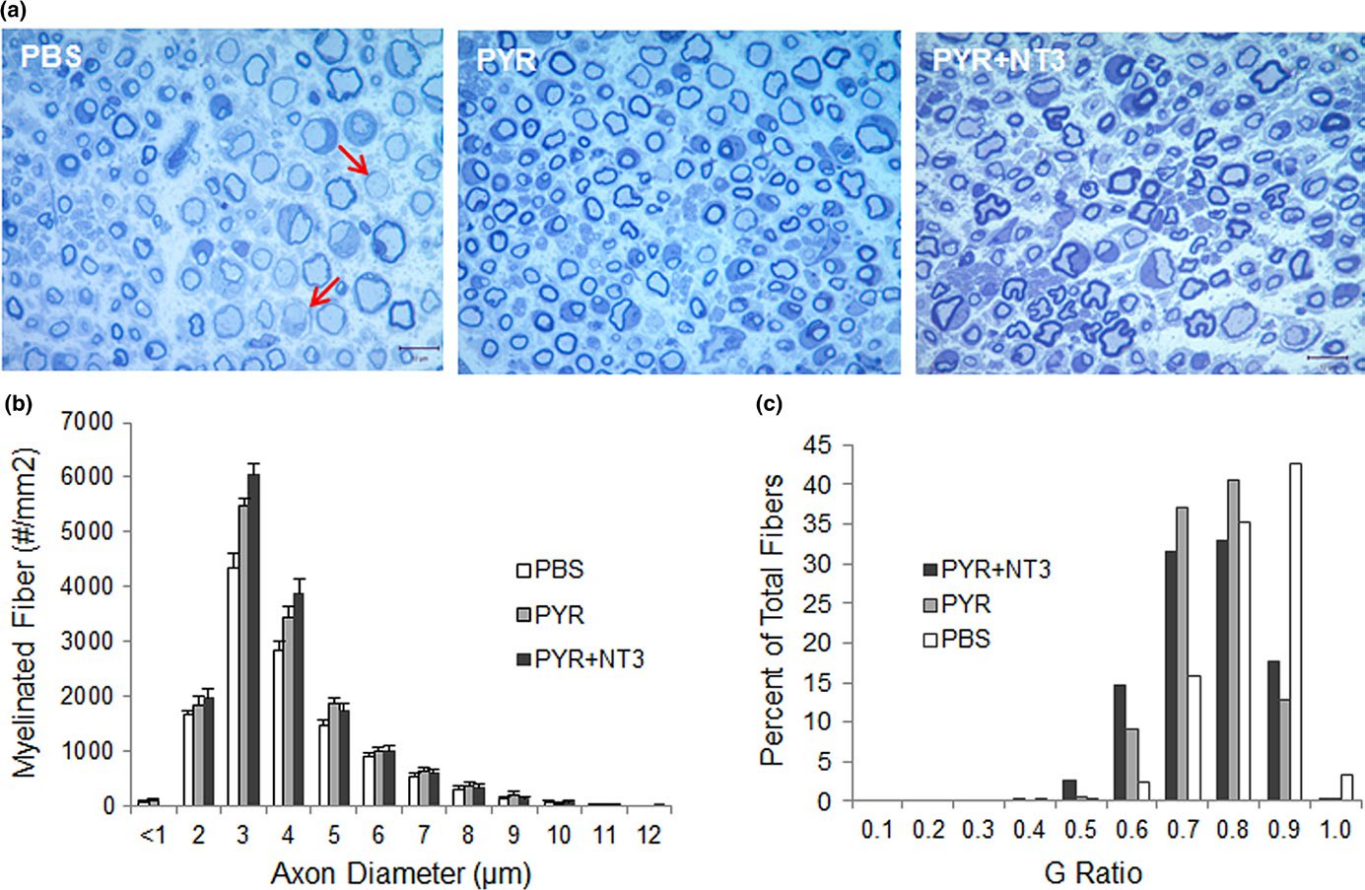
One‐micrometer thick toluidine blue‐stained representative cross sections of sciatic nerves (a) from control Tr**^J^** mice (injected with PBS), treated with pyruvate (PYR) and pyruvate plus AAV1.NT‐3 gene therapy (PYR + NT‐3) at 16 weeks. Thinly myelinated and naked axons are indicated with arrows in PBS‐treated nerves. PYR alone and combinatorial therapy result in an increase of axons with thicker myelin and an apparent increase in the small myelinated fibers. Composite histograms showing myelinated fiber distribution in the sciatic nerves (b) from control Tr**^J^** mice (PBS), treated with PYR and PYR + NT‐3 at 16 weeks showing an increase in the subpopulation of axons <4 μm in diameter in the PYR and PYR + NT‐3 treatment groups compared to PBS‐control. G ratio distribution of fibers in the sciatic nerves from Tr^J^ mice treated with PBS, PYR, or PYR + NT‐3 for 16 weeks (c). A shift toward increased percent of fibers with smaller G ratio (thicker myelin) is more pronounced with the combination treatment

Sixteen weeks of pyruvate supplementation resulted in significant histopathological improvements in the Tr^J^ sciatic nerves. Cross sections from the mid‐sciatic nerves revealed an apparent increase of the MF population in the pyruvate alone and in the combinatorial therapy cohorts compared to the untreated PBS group (Figure 2a). The quantitative studies revealed a significant increase in the MF densities in both treatment groups compared to untreated controls (Table 1). Combinatorial therapy compared to pyruvate alone did not result in a significant increase in the mean MF density, although the myelinated axon diameter distribution histograms showed a more prominent increase in a subpopulation of fibers with axon diameter between 2‐ and 4 µm (6,061.7 ± 173.4 vs. 5,490.5 ± 138.9/mm^2^; *p* = 0.0278) compared to pyruvate alone group (Figure 2b).

**Table 1:** Myelinated fiber density in Tr^J^ sciatic nerves

Groups	*N*	Myelinated fiber density	*p* Value
At 16 weeks			
Intact			
PYR	6	15,000.2 ± 878.3	0.0002
AAV1.NT−3 + PYR	6	15,782.0 ± 1,099.4	0.0001
PBS	6	12,377.9 ± 918.3	
At 20 weeks			
Intact			
PYR	5	16,583.2 ± 748.6	0.0271
Untreated	3	13,600.0 ± 325.4	
Regenerating			
PYR	5	18,258.9 ± 1,050.9	0.0075
Untreated	3	13,728.4 ± 1,469.8	

**Figure 3:**
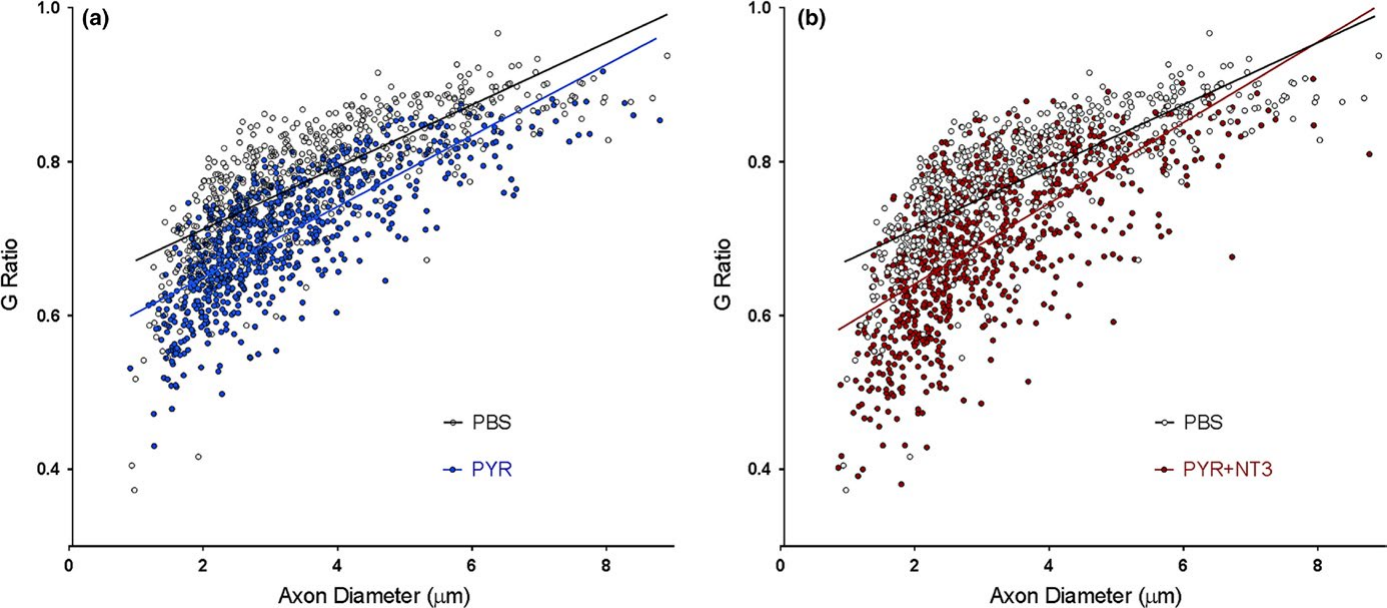
G ratios (axon diameter/fiber diameter) shown as scatterplot against respective axon diameter with linear regression. (a) PBS vs. PYR, the slopes are significantly different between the two groups: PBS, 0.04047 ± 0.001422 (*r* ^2^  = 0.5884); PYR, 0.04612 ± 0.001341 (*r* ^2^  = 0.6076). Linear regression, *p* < 0.0038. (b) PBS vs. PYR + NT3, the slopes are significantly different between the two groups: PBS, 0.04047 ± 0.001422 (*r* ^2^  = 0.5884); PYR + NT3, 0.05245 ± 0.001944 (*r* ^2^  = 0.4464). Linear regression, *p* < 0.0001

Improvements in myelin thickness in the treated cohorts were also evident (Figure 2a). G ratio (axon diameter/fiber diameter) determinations of the MFs in sciatic nerves showed an increase in myelin thickness indicating that pyruvate supplementation is partially improving the hypomyelination/amyelination state of trembler pathology. Combining pyruvate with AAV1.tMCK.NT‐3 gene therapy provided additional improvement in G ratio compared to pyruvate alone cohort with a further shift to smaller G ratios, thicker myelin (Figure 2c). The mean G ratio from sciatic nerve in the control Tr^J^ is 0.77 ± 0.003, significantly greater than that obtained from the WT (not shown, Sahenk et al., 2010; 0.66 ± 0.002, *p* < 0.0001), reflecting the hypomyelination state in the Tr**^J^** model. Both in combinatorial and pyruvate therapy cohorts, the percent of fibers having mean G ratio between 0.6 and 0.7 were substantially more than the untreated control Tr**^J^** nerves suggesting that these nerve fibers might be functionally meaningful. G ratios, shown as scatterplot against respective axon diameter with linear regression revealed significantly decreased G ratio, that is, improved myelination with pyruvate supplementation compared with untreated (PBS) trembler nerves and that this improvement was more significant with the combinatorial therapy (Figure 3a,b).

**Figure 4:**
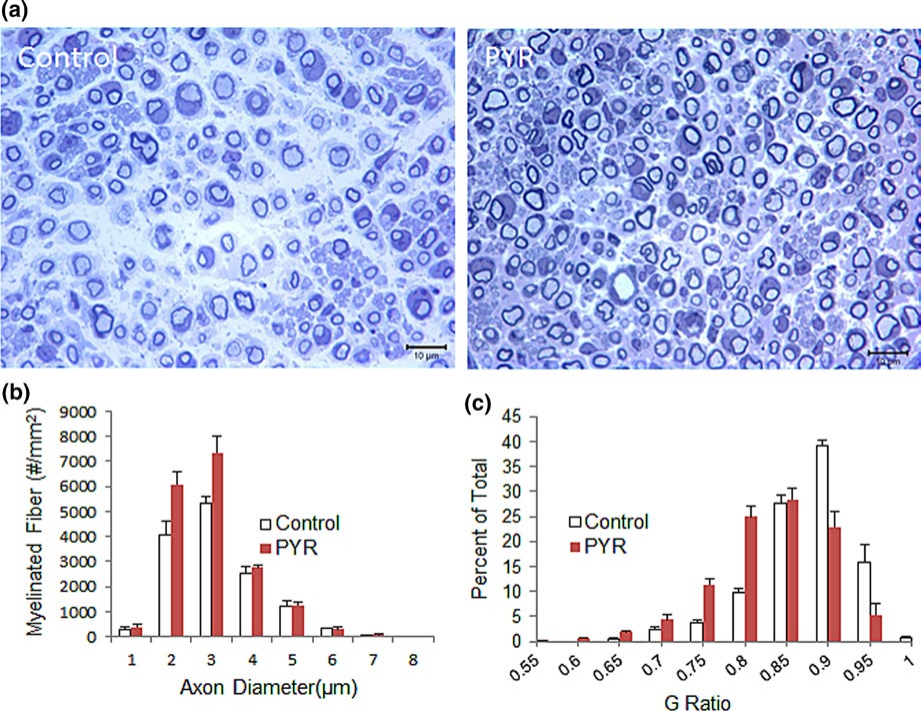
One‐micrometer thick toluidine blue‐stained representative cross sections (a) of regenerating sciatic nerves from Tr**^J^** control mice and treated with pyruvate (PYR) at 20 weeks. An apparent increase in the small myelinated fibers in regenerating nerves from PYR treated group is present. Composite histograms showing myelinated fiber distribution in the regenerating sciatic nerves (b) from Tr**^J^** mice at 20 weeks post‐PYR therapy showing an increase in the subpopulation of axons <4 μm in diameter in pyruvate group compared to PBS‐control. G ratio distribution of fibers (c) in the regenerating sciatic nerves from Tr**^J^** mice showing a shift toward increased percent of fibers with smaller G ratio (thicker myelin) in the PYR group

In another cohort, the efficacy of pyruvate supplementation upon regeneration was assessed using the sciatic nerve crush paradigm at 20 weeks post‐crush time point, as we had carried out in our previous studies (Sahenk et al., 2014, 2010 ). In the treated group, microscopic examination of cross sections of sciatic nerve segments showed an increase in the number of MFs in the regenerating and the contralateral intact sciatic nerves compared to the untreated Tr**^J^** counterparts. A notable increase of MFs in the regenerating nerve segments was evident in pyruvate‐treated Tr^J^ mice compared to regenerating control Tr**^J^** nerves (Figure 4a). Quantitative studies confirmed these observations and revealed statistically significant increases in MF densities in the intact and regenerating sciatic nerves at 20 weeks post‐crush time point (Table 1). Figure 4b shows the composite histograms generated from the regenerating segments approximately 3 mm distal to the crush site from pyruvate‐treated‐ and control Tr^J^ mice. We previously showed a defect in regeneration in trembler nerves, supported with a significant decrease in the number of MFs compared to WT counterparts (Sahenk et al., 2010, 2005 ). The pyruvate supplementation improved the MF densities of regenerating trembler nerves significantly (Table 1), and similar to the intact nerves, we found the increase in MF densities in the regenerating nerves was most prominent for those fibers with axonal diameter between 2‐ and 4 µm at this time point. G ratio determinations from the pyruvate‐treated regenerating nerves also showed a shift to the thicker myelin compared to untreated regenerating trembler nerves (Figure 4c).

When the mean MF density from the intact contralateral nerves at 20 weeks treatment cohort (16,583.2 ± 748.6/mm^2^, *n* = 5) was compared to that obtained at 16 weeks (15,000.2 ± 878.3/mm^2^, *n* = 6; *p* = 0.0652), we observed an additional increase in the MF density suggesting that continuation of pyruvate supplementation may provide added benefit (Supporting Information Figure S2). While the mean of total MF densities was not statistically significant (*p* = 0.0652), the improvement in the subpopulation of MFs with axon diameter between 2‐ and 4 µm was highly significant (*p* < 0.002). Collectively, these results show that 16 weeks of pyruvate supplementation was sufficient to maintain CMAP amplitudes and increased myelinated fiber densities and myelin thickness and improved motor performance of Tr**^J^**mice. Combining exogenous pyruvate with AAV1.tMCK.NT‐3 gene therapy provided additional benefits showing significantly improved CMAP amplitudes and nerve conduction velocities as well as myelin thickness compared to pyruvate alone cohort. As we observed with NT‐3 gene therapy (Sahenk et al., 2014), duration of treatment, prolonged pyruvate supplementation augmented the therapy efficacy.

### 3.3 Expression of ER and mitochondrial stress markers after pyruvate treatment

**Figure 5:**
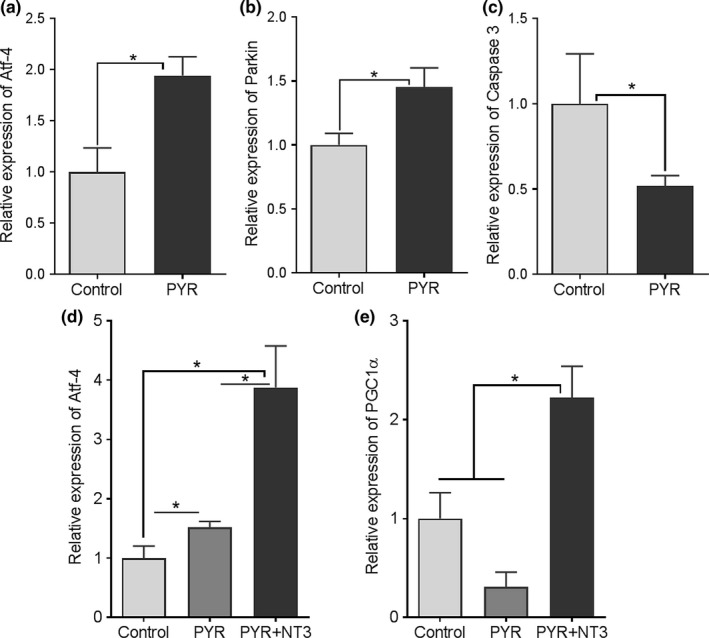
Relative expression levels of Atf4 (a, d), Parkin (b), and Caspase 3 (c) and PGC1α (e) in sciatic nerve samples collected at the end point. GAPDH was used as housekeeping gene in the analyses. Results shown are mean ± *SEM* unless otherwise indicated; *n* = 4–6 each group. Statistical significance: Student’s *t* test (for a–c) and one‐way analysis of variance followed by Tukey's multiple comparison test (for d and e); *p < 0.05. Control stands for samples from untreated Tr**^J^**mice

Previous studies have shown that both overexpression of WT PMP22 and the mutant Tr**^J^** protein form a complex with calnexin, a Ca^2+^‐binding chaperone, that contributes to endoplasmic reticulum (ER) retention (Dickson et al., 2002). Accumulation of mutant PMP22 protein in Tr**^J^** SCs can also potentially trigger ER stress, resulting in SC cell death by apoptosis, and subsequently causing peripheral neuropathy (Khajavi et al., 2007; Sancho, Young, & Suter, 2001). Activating transcription factor 4 (Atf4) was shown to be a major player in coping with ER stress by regulating the glucose homeostasis and inhibiting the bulk protein synthesis (Wek & Cavener, 2007). Atf4 expression is controlled by eIF2/protein *kinase* RNA‐like endoplasmic reticulum *kinase (*elF2α/PERK). eIF2a phosphorylation causes a reduction in global protein synthesis while allowing the translation of selected genes including ATF4, aiding cell survival and recovery (Lu, Harding, & Ron, 2004). PERK also directly stabilizes ER–mitochondrial contacts and promotes formation of ER–mitochondrial interactions through the transcriptional upregulation of Parkin (Lin & Popko, 2009). The stress‐induced upregulation of parkin is mediated by ATF4, and parkin has a role in the interorganellar cross talk between the ER and mitochondria to promote cell survival under stress (Bouman et al., 2011). For all these ATP dependent regulations, sustaining ATP by metabolizing pyruvate or its derivatives might play crucial role in resolving ER stress. We therefore investigated if pyruvate therapy alters the expression of ER stress markers in trembler nerves by determining the expression levels of these key stress markers, Atf4, Parkin, and Caspase‐3. Real‐time PCR on fresh frozen sciatic nerve samples at 16 weeks revealed that pyruvate treatment increased the expression levels of Atf4 and parkin which correlated with reduced ER stress and reduced Caspase‐3 expression compared to untreated trembler nerves (Figure 5a–c). Moreover, compared to pyruvate alone, combining pyruvate with the AAV1.tMCK.NT‐3 gene therapy had an additive effect in coping with ER stress and significantly increased Atf4 expression levels (Figure 5d).

We also examined whether the improved myelination in the trembler nerves was associated with altered expression levels of the mitochondrial biogenesis marker peroxisomal proliferator activator receptor‐γ coactivator‐1α (PGC1α). Mitochondrial biogenesis may increase in cells under conditions such as proliferation, differentiation/growth, or cell stress. PGC1α was shown to play a role in postnatal myelination and that deficient PGC1α activity in oligodendrocytes may contribute to abnormal myelination in some neurodegenerative disease models (Xiang et al., 2011). Figure 5e shows the expression levels of PGC1α in the sciatic nerve samples from the Tr**^J^** cohorts at 16 weeks, treated with pyruvate alone or pyruvate plus AAV1.tMCK.NT‐3 gene therapy compared to the untreated controls. We found that pyruvate had not affect the PGC1α expression similar to the study in C2C12 cells in which pyruvate‐induced mitochondrial protein expression occurred by a PGC1α‐independent mechanism (Wilson, Yang, Szustakowski, Gullicksen, & Halse, 2007). On the other hand, the trembler nerves from combinatorial therapy cohort showed increased PGC1α expression compared to pyruvate alone or the untreated trembler nerves suggesting that this effect is induced by NT‐3 (Yalvac et al., 2018; Figure 5e).

## 4 DISCUSSION

In this study, we sought to evaluate the potential therapeutic use of oral pyruvate supplementation in the Tr^J^ mouse model, alone and in combination with AAV1.NT‐3 gene therapy using electrophysiological, functional, and histopathological studies. This approach was based on the assumption that exogenous pyruvate should provide readily attainable ATP to energy deprived axons and to decrease ER stress in the mutant/dysfunctional SCs of trembler nerves.

The maintenance of bioenergetics homeostasis is essential for cell survival, and this is particularly important for structural and functional integrity of distal axons due to its unique morphology, therefore requiring extraordinary demand for ATP to support energy consuming activities, including axoplasmic transport and generation of ion gradients. Intuitively, it may be concluded that for whatever the underlying genetic or acquired causes are once a point of no return is reached, energy deprived axons will have the same fate, Wallerian‐like degeneration. Recent studies are now emphasizing axonal pathology as an early event that leads to axon degeneration and pathological hallmark in a wide‐ranging acquired and hereditary neurodegenerative disorders, especially in those primarily affecting the glia (Beirowski, Babetto, & Wrabetz, 2016). In fact, our earlier studies and others have brought secondary axonal pathology into attention in disorders with SC‐specific genetic defects (de Waegh & Brady, 1991; de Waegh et al., 1992; Griffiths et al., 1998; Sahenk, 1999; Sahenk & Chen, 1998; Sahenk et al., 1999; Sahenk, Chen, & Freimer, 1998). Independent of myelin itself and its insulator role in saltatory conduction, several recent studies have provided evidence for a key function of glia, that is metabolic support of axons (Nave, 2010). According to this premise, metabolic deficits in SCs or oligodendrocytes, or impaired metabolite transport, are thought to account for axonal degeneration (Beirowski et al., 2014; Lee et al., 2012). Intermediate products of glycolysis such as lactate in oligodendrocytes appear to support CNS axons (Funfschilling et al., 2012; Lee et al., 2012). Furthermore, a genetic defect‐induced dysfunction in CS may compromise this energy support to axon due to ER stress resulting from accumulation of mutant protein as seen in Tr**^J^** or in the CMT1A rodent models resulting from overexpression of normal PMP22 as aggresomes (Clayton & Popko, 2016; Fortun et al., 2006; Liu, Yamauchi, & Shooter, 2004; Volpi, Touvier, & D'Antonio, 2016). This compromise was also shown in myelin protein zero (P0) mutant protein‐induced ER stress (Wrabetz et al., 2006). Therefore, the central notion in our studies was to restore the bioenergetics homeostasis of axons and SCs by pyruvate supplementation, simply providing readily available natural metabolic fuel to mitochondria for ATP production and also take advantage of its antioxidant properties.

A glycolytic end product, pyruvate enters mitochondria via the inner membrane monocarboxylate transporter and assumes a central role in cellular energy production. Due to its α‐keto‐carboxylate structure, pyruvate also has antioxidant properties and can directly accomplish radical scavenging as shown in neutralization of peroxides and peroxynitrite, (Mallet, Sun, Knott, Sharma, & Olivencia‐Yurvati, 2005). Another metabolic effect of exogenous pyruvate is to improve the mitochondrial redox states (NAD/NADH) which facilitate oxidative phosphorylation and the glutathione redox cycle (NADP/NADPH) for counteracting oxidative stress (Alvarez, Ramos, Ruiz, Satrustegui, & Bogonez, 2003; Kashiwagi et al., 1997; Mongan et al., 2002; Moro, Ghavim, Harris, Hovda, & Sutton, 2016; Wang et al., 2007). Pyruvates, including sodium, methyl and ethyl derivative of pyruvic acid, have been shown to be protective in various in vitro and in vivo models of oxidative stress (Das, 2006; Dobsak et al., 1999; Mallet et al., 2005; Paromov et al., 2011). Anti‐cell death and anti‐inflammatory mechanisms were shown in play for its protective function in various preclinical disease models (Kao & Fink, 2010; Shen et al., 2010; Wang et al., 2007; Wilson et al., 2007). Moreover, in recent studies using a novel ex vivo approach for monitoring mitochondrial dynamics within axons, NAD+ and pyruvate not only protected mitochondria from oxidative damage, maintained its transport, but also prevented subsequent degeneration of axons (Bros, Millward, Paul, Niesner, & Infante‐Duarte, 2014).

Key findings in our study are that pyruvate supplementation improved regeneration and myelination of trembler nerves and that there is a potentiating effect of the combinatorial therapy when we combined exogenous pyruvate with NT‐3 gene therapy simultaneously. This approach resulted in further improvements in a subpopulation of MF densities and myelin thickness, improved sciatic nerve conduction velocity and CMAP amplitudes in trembler nerves. The capacity of NT‐3 targeting the translational machinery to stimulate myelin protein synthesis was previously shown in oligodendrocyte primary cultures (Coelho, Yuelling, Fuss, & Sato‐Bigbee, 2009), and the same mechanism is likely to take place in the SCs. In fact, NT‐3 is capable of activating Akt/mTORC1 pathway, and in a recent study, we showed a novel effect of NT‐3, its ability to directly influence the protein synthesis and metabolic remodeling in the neurogenic muscle from Tr**^J^** mouse (Yalvac et al., 2018). Of note, while the NT‐3‐induced fiber size increase was most prominent for the fast twitch glycolytic fiber population, exogenous pyruvate had a different effect, increased the fiber diameter of slow twitch oxidative fibers preferentially in the Tr**^J^** mouse (unpublished observations). When compared to pyruvate alone, failure to show an increase in the mean MF density with combinatorial therapy may suggest an apparent discrepancy between grip strength improvement and CMAP/nerve density changes*.* It is, however, likely that the functional tests were reflection of improvements in multiple muscle groups and other factors including improved force generation related to muscle diameter increase (Rodino‐Klapac et al., 2013) and increased axonal sprouting in muscle, both of which can occur from NT‐3 effect.

In our study, we also presented evidence for ER protective effect of pyruvate. Sciatic nerve samples at 16 weeks of pyruvate treatment increased the expression levels of Atf4 and parkin compared to untreated trembler nerves. Increased expression levels of Atf4 and parkin were shown to correlate with reduced ER stress and reduced Caspase‐3 expression (Lin & Popko, 2009; Wek & Cavener, 2007). Studies in *PMP22* transgenic mutants and Tr**^J^**mice have shown that a substantial increase in SC apoptosis occurs during the postnatal period compared with the wild type and that continues throughout the adult life of the animals (Sancho et al., 2001). Therefore, these properties of pyruvate may contribute to effective regeneration by increasing available SC pool compared to untreated trembler nerves as shown in crush experiments. Moreover, compared to pyruvate alone, combining pyruvate with NT‐3 gene therapy had an additive effect in coping with ER stress and significantly increased Atf4 expression levels. Further studies are needed to establish causal relationship between these correlative markers and the improved outcome measures with pyruvate alone and combined treatment with NT‐3, in particular, the role of NT‐3 on the parkin expression.

It is likely that all above discussed mechanisms of the action of pyruvate are important in rescuing trembler pathology. We believe exogenous pyruvate alone or as adjunct therapy will have significant clinical implications given that the tool for energy replacement through this treatment as an oral agent in patients is simple and straight forward. To start with, this initial preclinical study validating efficacy of exogenous pyruvate in this CMT model should lead to confirmatory studies with additional rigor including blinding methods, power calculations with additional outcome measures at multiple doses and treatment durations which can be extrapolated from previous reports on patients. Design of blinded clinical trials using sodium pyruvate will not be difficult given its mild side effects profile (Fujii et al., 2014). Pyruvate has been administered to patients for a variety of conditions (Dijkstra et al., 1984; Fujii et al., 2014; Giannelli et al., 1976; Hermann, 2001; Hermann et al., 1999; Koga et al., 2012; Olivencia‐Yurvati et al., 2003; Tanaka et al., 2007) and deserves considerations for peripheral neuropathies, disorders with a limited repertoire of treatment options.
