# Supplementary figures and images for: Efficacy of exogenous pyruvate in TremblerJ mouse model of Charcot‐Marie‐Tooth neuropathy
Source: Brain Behav. 2018 Sep 21;8(10):e01118. doi: 10.1002/brb3.1118 (PMC6192403; doi:10.1002/brb3.1118)

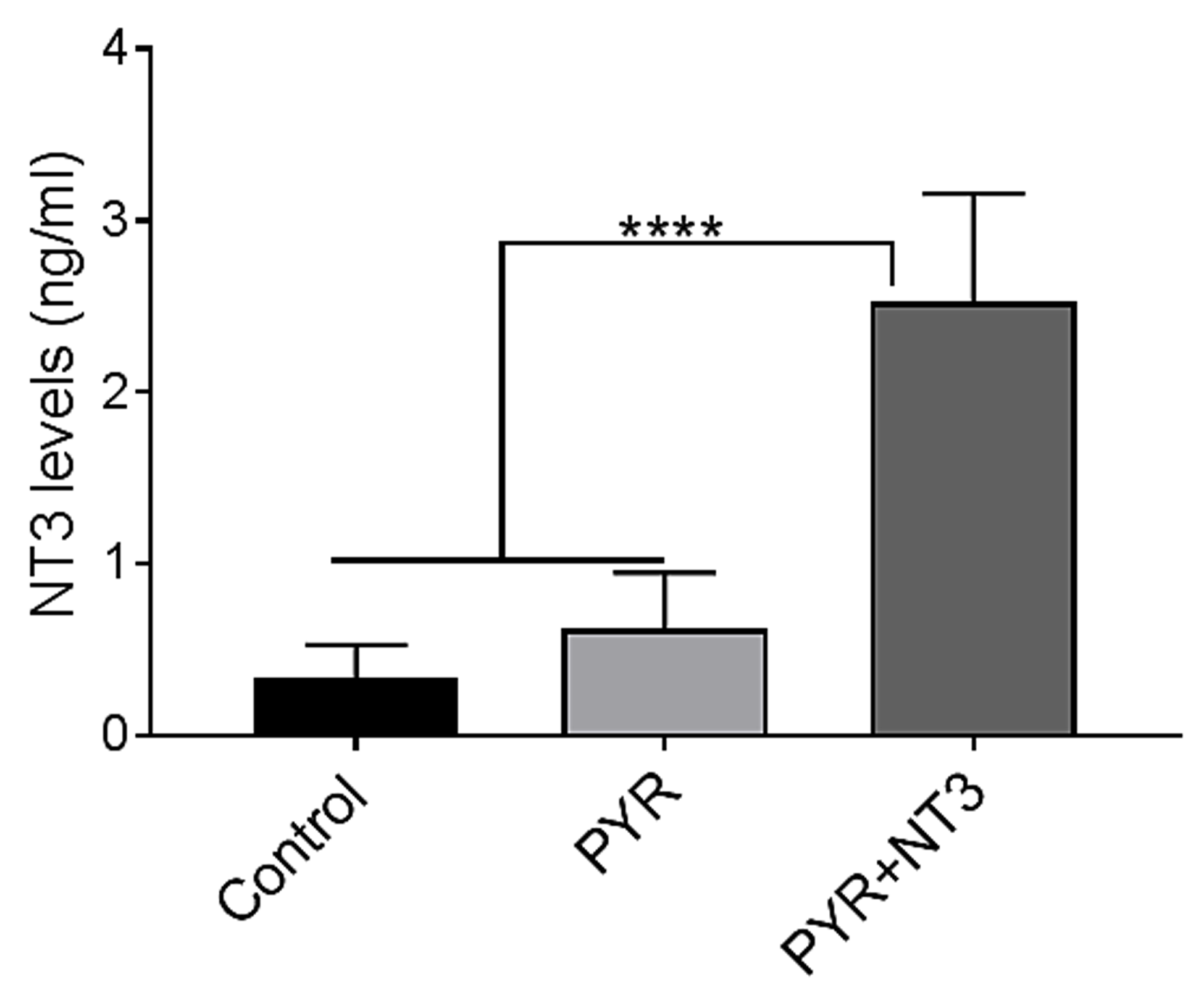

Supplement: Supplementary file 1 [file BRB3-8-e01118-s001.tif]

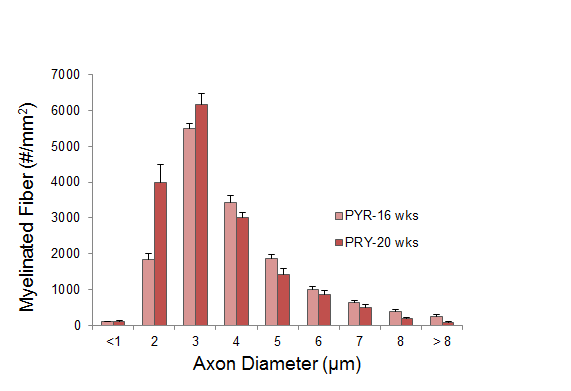

Supplement: Supplementary file 2 [file BRB3-8-e01118-s002.tif]
